# Tracking Low-Copy Transcription Factors in Living Bacteria: The Case of the *lac* Repressor

**DOI:** 10.1016/j.bpj.2017.02.028

**Published:** 2017-04-11

**Authors:** Federico Garza de Leon, Laura Sellars, Mathew Stracy, Stephen J.W. Busby, Achillefs N. Kapanidis

**Affiliations:** 1 Gene Machines Group, Clarendon Laboratory, Department of Physics, University of Oxford, Oxford, United Kingdom; 2 School of Biosciences, University of Birmingham, Edgbaston, Birmingham, United Kingdom

## Abstract

Transcription factors control the expression of genes by binding to specific sites in DNA and repressing or activating transcription in response to stimuli. The *lac* repressor (LacI) is a well characterized transcription factor that regulates the ability of bacterial cells to uptake and metabolize lactose. Here, we study the intracellular mobility and spatial distribution of LacI in live bacteria using photoactivated localization microscopy combined with single-particle tracking. Since we track single LacI molecules in live cells by stochastically photoactivating and observing fluorescent proteins individually, there are no limitations on the copy number of the protein under study; as a result, we were able to study the behavior of LacI in bacterial strains containing the natural copy numbers (∼40 monomers), as well as in strains with much higher copy numbers due to LacI overexpression. Our results allowed us to determine the relative abundance of specific, near-specific, and non-specific DNA binding modes of LacI in vivo, showing that all these modes are operational inside living cells. Further, we examined the spatial distribution of LacI in live cells, confirming its specific binding to *lac* operator regions on the chromosome; we also showed that mobile LacI molecules explore the bacterial nucleoid in a way similar to exploration by other DNA-binding proteins. Our work also provides an example of applying tracking photoactivated localization microscopy to studies of low-copy-number proteins in living bacteria.

## Introduction

Transcription factors (TFs) repress or activate transcription by directly controlling gene expression. One of the best characterized TFs is the *Escherichia coli lac* repressor (LacI), which controls the ability of a cell to uptake and metabolize lactose. In the absence of lactose, LacI specifically binds a control region in DNA (the *lac* operator) and blocks transcription of the *lac* operon, thus repressing the genes for lactose utilization. In the presence of lactose, LacI binds to allolactose (a metabolic intermediate of lactose) and shows markedly reduced affinity for its operator (1).

Understanding gene regulation and its control elements can aid systems biology efforts to create models of larger systems. Furthermore, understanding control systems based on TFs will extend the available toolboxes for synthetic gene expression. TFs can also be a tool for imaging, as shown with the use of fluorescent repressor/operator systems (FROSs) to tag chromosomal locations (2, 3). Since LacI is one of the best characterized DNA-binding proteins, further research into its intracellular mobility, molecular interactions, and subcellular distribution should advance our understanding of many other proteins interacting with DNA.

The mechanism by which LacI finds its target operator (a 21-bp-long DNA sequence) among the long stretches of non-specific chromosomal DNA (∼4.5 Mbp for *E. coli*) has been studied extensively (4, 5, 6, 7, 8, 9). Initial biochemical work showed that LacI binds to its specific site at rates faster than expected from free three-dimensional (3D) diffusion (4, 10); such results led to the proposal of target location through facilitated diffusion, whereby DNA-binding proteins utilize a combination of 3D diffusion, non-specific DNA binding, and one-dimensional (1D) sliding on DNA to locate their target swiftly. More recently, single-molecule studies, both in vitro and in vivo, have provided strong support for the facilitated diffusion model. In vitro studies have shown that LacI can bind and slide on DNA with a 1D diffusion coefficient of ∼0.02 *μ*m^2^ s^−1^ (8, 9). However, such techniques stretch out DNA from its native form, ignoring any effects of DNA deformation and bending on LacI sliding; further, sliding also depends on salt concentrations and buffer conditions used in vitro, which cannot replicate the environment encountered in living cells.

Single-molecule measurements in individual living cells can address many of the limitations of in vitro work, since such measurements go beyond the ensemble averaging of cell populations, retain molecular resolution, and uncover cell-to-cell heterogeneity (11). Recent experiments have examined the behavior of single fluorescently labeled LacI molecules inside live *E. coli* cells. Using strains with artificially low copy numbers (∼7 monomers/*lacI* gene), it became possible to track single LacI molecules in live *E. coli* (7, 12, 13), and determine the effective diffusion coefficient for the combination of non-specific binding and free diffusion (0.4 *μ*m^2^ s^−1^). These results led to the conclusion that LacI spends an estimated ∼87% of its time non-specifically bound to DNA (7), where it slides for an average of ∼45 bp (12). However, to date, no such work has been done on strains with the native LacI copy numbers (∼40 monomers; see (14)).

In addition to diffusion, the spatial distribution of LacI is also relevant to target location, since any LacI concentration gradients will influence the target search times and the status of repression (15, 16, 17). Previous work using fixed cells showed that the subcellular spatial distribution of LacI is non-uniform and depends on growth rates and the position of the transcribed gene (15). In contrast, live-cell work showed that LacI does not maintain high local concentrations (13); as a result, it is currently unclear whether endogenously expressed LacI is uniformly distributed in the cell.

Here, we study the LacI intracellular mobility and spatial distribution of LacI in live bacteria using photoactivated localization microscopy (PALM) combined with single-particle tracking (tracking PALM) (18, 19, 20, 21, 22). Since we track single LacI molecules in live cells by stochastically photoactivating and observing fluorescent proteins individually, we have no limitations on the copy number of the protein under study; as a result, we were able to study the behavior of LacI in bacterial strains that contain the natural copy numbers for LacI, as well as much higher copy numbers due to LacI overexpression. Our results allowed us to determine the relative abundance of specific, near-specific, and non-specific DNA binding modes of LacI in vivo, showing that all these modes are operational in cells. Further, we examined the spatial distribution of LacI in live cells, confirming that its specific binding is indeed to *lac* operators on the chromosome; we also showed that the mobile LacI is exploring the bacterial nucleoid, similar to the way other DNA-binding proteins explore it. Our work provides procedures and recommendations for applying tracking PALM to studies of low-copy-number proteins in living bacteria.

## Materials and Methods

### Strain construction

Strains were constructed as published in (23). Six LacI operator DNA sites (5′-AATTGTGAGCGGATAACAATT-3′) were inserted adjacent to the araBAD promoter in a strain that also had a chromosomal LacI::GFP fusion, LR06. The 6x lac operator sequence, as well as the DNA segment of the 6x lac operator sequence adjacent to 20x MalI operator sequences, is provided in the Supporting Material.

### Cell culture

A plate was streaked and placed overnight at 37°C, and a single colony was placed into a culture tube in 5 mL of lysogeny broth (LB). The culture was grown for 4 h at 37°C. The culture was diluted 10,000 times in M9 medium supplemented with MEM amino acids and L-proline, MEM vitamins, and 0.2% glycerol. The diluted culture was grown overnight at 37°C. The next day, the cells were diluted to ∼0.025 OD_600_ in the M9 medium just described and grown at 37°C for ∼2 h until they reached an OD_600_ of 0.1. The cells were then concentrated by centrifugation and immobilized in 15 *μ*L wells with 1% polyethylenimine. Induction of cells with isopropyl *β*-D-1-thiogalactopyranoside (IPTG) was done by mixing 1 mM of IPTG in the M9 media. To induce expression on the slide, the cells were washed with the M9 minimal media supplemented with 1 mM IPTG.

### Imaging

A bespoke wide-field microscope was used to image PAmCherry fusions using a 405-nm photoactivation laser and a 561-nm excitation laser (iChrome MLE-LFA, TOPTICA Photonics, Rochester, NY). The 405-nm laser was controlled between 0 and ∼1 W cm^−2^ to control the photoactivation rate; the 561-nm laser was kept constant at 200 W cm^−2^. A dichroic mirror (ZT405/488/561rpc, Chroma, Foothill Ranch, CA) and an emission filter (ZET405/488/561NF, Chroma) were used to filter the emission. The objective is a 100× oil-immersion microscope (UPLANSAPO, 100×, Olympus, Center Valley, PA) and the camera is an EMCCD (iXon Ultra, 512 × 512, Andor, Belfast, United Kingdom) with 15.48 ms/frame for the tracking-PALM experiment and 100 ms/frame for the FROS foci imaging. We use a high-inclined illumination (HiLo) excitation mode, ensuring illumination of the entire cell and minimal loss of particles moving out of focus.

For the FROS work, a different bespoke wide-field microscope was used to image PAmCherry fusions at 0–1 W cm^−2^ with a 405 nm photoactivation laser (MLL-III-405, 100 mW, CNI Laser, Madison Heights, MI), a 115 W cm^−2^ 475 nm (70 mW Stradus, Vortran Laser Technology, Sacramento, CA) and a 400 W cm^−2^ 561 nm excitation laser (SLIM-561, 200 mW, Oxxius, Lannion, France). The 405 nm laser was controlled between 0 and 10 *μ*W to limit the photoactivation rate; the 475 nm laser was kept at a constant of 1 mW; and the 561 nm laser was kept at a constant of 3.5 mW. A dichroic mirror (ZT405/473/561rpc, Chroma) and a notch filter (ZET405/473/561NF, Chroma) were used to filter the emission. The objective is a 100× oil-immersion microscope (UPLANSAPO, 100×, Olympus) and the camera is an EMCCD (iXon 897, 512 × 512 pixels, Andor) set to 15.26 ms/frame for the tracking-PALM experiment and 100 ms/frame for the FROS foci imaging.

### Analysis

We used MicrobeTracker (24) to segment cells and analyze tracks on a per-cell basis. To track the single molecules, we used custom-written code in MATLAB. We localized the molecules using elliptical Gaussian fitting. Localizations that were consecutively within a radius of 0.57 *μ*m were linked; we also allowed for the molecules to disappear (due to blinking or defocusing) for one frame. The mean-square displacement (MSD) of each track was calculated to analyze the diffusion of the molecules. The apparent diffusion coefficient was calculated as

$$D^{*}=\frac{\text{MSD}}{4\Delta t},$$

where *D^∗^* is the apparent diffusion coefficient (*μ*m^2^/s) and Δ*t* is the time lag. The *D^∗^* includes localization error in its calculation. The bound population functional form comes from an experimental control that comprises bacterial cells containing a DNA-bound DNA polymerase I tagged with PAmCherry. The apparent motion of bound molecules is mainly due to localization uncertainty, which manifests itself as a shift to the right in the *D*^∗^ value by *σ*_loc_^2^ Δ*t*^−1^; hence, immobile molecules appear to have a *D*^∗^ value of ∼0.1 *μ*m^2^ s^−1^ due to the localization uncertainty of ∼40 nm.

To measure the apparent coefficient of an immobile molecule, cells were fixed in 2.5% paraformaldehyde (22), and a threshold was defined for the molecules that are bound: *D*^∗^_thres_ = 0.1 *μ*m^2^/s; any molecules below this *D*^∗^ value are deemed to be immobile. The radial distribution was constructed with radial binning at *dr* = 40 nm.

### Fitting *D*^∗^ distributions

As described in (25), (26), and (27), we find the diffusion coefficient constant, $D_{1}^{*}$, by fitting the probability density of the *D*^∗^ distribution of a sample with an analytical equation for the constant, $D_{1}^{*}$. We used tracks that had a minimum of four steps and truncated longer tracks at four steps. This generates an analytical expression for the single-mode case:

fD∗(x;D1∗)=(4D1∗)4x3e−4xD1∗6,

where *x* is the empirical *D*^∗^ data. The different modes of LacI binding can be separated into two modes, bound and mobile. A second mode is then introduced, and the analytical equation becomes

fD∗(x;D1∗,D2∗,A1,A2)=A1(4D1∗)4x3e−4xD1∗6+A2(4D2∗)4x3e−4xD2∗6,

where the variables $D_{1}^{*}$ and $D_{2}^{*}$ are the apparent diffusion coefficients for each of the two modes and *A*_1_ and *A*_2_ are the respective fractions contributing to the *D*^∗^ distribution.

## Results

### Diffusion analysis of single LacI molecules using tracking PALM

In *E. coli*, tracking PALM has been used to analyze several in vivo processes related to nucleic acid metabolism, such as stringent response (21), DNA repair (22), and gene transcription (25). All of these processes were studied by tracking proteins present in high copy numbers (100–10,000) in each cell. Here, we apply tracking PALM to the study of LacI, a transcriptional repressor that has low copy numbers in each cell (∼40 monomers per cell). Since LacI has a well-studied function and intracellular mobility, it is ideal as a model protein for establishing methods for studying TFs with low to moderate copy numbers per cell (10–100 monomers); indeed, many TFs in bacteria fall in this copy number range.

To draw a parallel to the copy numbers in previous studies and gain more statistical information per cell, we first studied cells with a high copy number for LacI. Specifically, we overexpressed LacI from a *lac* gene placed on a plasmid and regulated by two *lac* operators; overexpression was achieved by growing cells in the presence of 1 mM IPTG, a mimic of allolactose that binds LacI and prevents specific binding to the operator. To study the intracellular mobility of LacI under conditions where it is capable of specific DNA binding, we subsequently diluted IPTG (to 10 *μ*M) and added 1 mM 2-nitrophenyl *β*-D-galactopyranoside (ONPG), which outcompetes IPTG, binds LacI, and restores specific DNA binding of LacI to operator regions, as well as to any near-specific sites present in the bacterial chromosome.

**Figure 1:**
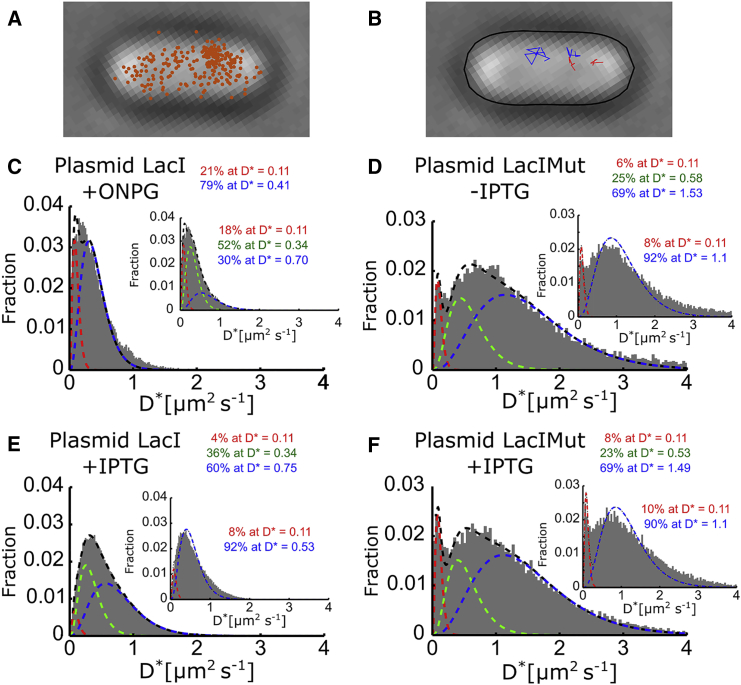
Single-molecule tracking of overexpressed LacI-PAmCherry and LacIMut-PAmCherry. We use tracking PALM to observe single LacI-PAmCherry molecules by photoactivating, then imaging using 15-ms exposures. (*A*) An example cell with all LacI localizations. (*B*) The same cell showing LacI tracks with four or more steps, classified as bound (*red*) and mobile (*blue*) by thresholding of their apparent diffusion, *D*^∗^. (*C*) *D*^∗^ histogram of the full-length LacI with ONPG (which was added to outcompete IPTG used to overexpress the LacI) fit with two species and three species (*inset*). (*D*) *D*^∗^ histogram for LacI without the DNA-binding domain fit with three species and two species (*inset*). Without the DNA-binding domain, LacIMut shows a reduced bound (or slow) fraction of molecules. (*E*). After IPTG is added, LacI shows reduced specific and non-specific binding to DNA. The three-species fit is shown together with the two-species fit (*inset*). (*F*) Addition of 1 mM IPTG to LacIMut does not change significantly its diffusion profile.

To perform tracking-PALM experiments on LacI, the repressor carried a C-terminal fusion with PAmCherry (28), a photoactivatable fluorescent protein that emits in the red spectrum. As shown for fusions of green fluorescent protein (GFP) derivatives with LacI (7), LacI-PAmCherry is expected to bind to a single operator as a dimer (not as a tetramer, as for wild-type LacI). Using photoactivatable proteins, we localized and tracked a single protein with ∼40 nm spatial precision, as in our previous work (22, 25). We photoactivated the pool of inactive LacI-PAmCherry using a 405-nm laser and tracked activated proteins using a 561-nm laser. This illumination scheme allowed us to observe a single LacI molecule until it bleached, with every frame (15 ms exposure) yielding a localization (Fig. 1 *A*). This series of steps was repeated until the entire pool of PAmCherry was tracked (Fig. 1 *B*). The density of observable LacI-PAmCherry in the cell was kept low (preferably a single photoactivated protein per cell) by controlling the photoactivation rate through regulation of the power of the 405-nm laser.

To measure the intracellular mobility of LacI, we used the MSDs of the tracked localizations to calculate the apparent diffusion coefficient (*D*^∗^) of each track with four steps (five localizations) or more (22). Since fluorescent proteins bleach quickly in tracking-PALM experiments, we collected an average of ∼3.5 localizations per track. With such short tracks, the *D*^∗^ values show large statistical uncertainty, which is evident in the spread of the *D*^∗^ distribution. By using tracks with a minimum number of steps (here, four steps), we reduced the spread of the *D*^∗^ distribution.

The *D*^∗^ distribution in the +ONPG sample reflects the LacI form able to bind DNA specifically (Fig. 1 *C*), as well as non-specifically (i.e., during its target search). Knowing the analytical expression for the distribution of *D*^∗^ values expected from a single diffusing species, we fit the data to the expected *D*^∗^ distribution with four steps (see Materials and Methods). A single-species fit to the *D*^∗^ distribution fails to describe it well (see Fig. S1 for fits and their residuals); this result was not surprising, considering the DNA-binding properties of LacI, which dictate that a fraction of molecules binds to DNA within our observation time for one molecule (75 ms).

We then fit the *D*^∗^ distribution to a two-species analytical model (Fig. 1 *C*, *red* and *blue dashed lines*; see Materials and Methods), which showed a substantial improvement over the single-species fit (based on the value and distribution of residuals; see Fig. S1 *E*) and yielded a fit that described LacI diffusion well. The first species corresponds to the fraction of bound LacI molecules, described by a distribution with a mean of *D*^∗^ = 0.11 *μ*m^2^ s^−1^ (Fig. 1 *C*, *red dashed line*); the non-zero *D*^∗^ value for these bound molecules represents the finite localization precision (corresponding to a localization error of ∼40 nm), and this has been confirmed for other DNA binding proteins using the same method and microscope (25). The second species corresponds to the mobile fraction of LacI and is described by an unconstrained *D*^∗^ distribution (Fig. 1 *C*, *blue dashed line*; note that the widths of the bound and mobile species differ, because the width depends on the mean, with a lower *D*^∗^ value corresponding to a smaller width). The bound species population accounts for ∼20% of the molecules, whereas the mobile species accounts for ∼80% of the molecules and has a mean *D*^∗^ of ∼0.4 *μ*m^2^/s.

We also explored a three-species model (Fig. 1 *C*, *inset*), which showed a small improvement in the fit quality (residuals were 6.2 × 10^−4^ for the two-species fit and 3.8 × 10^−4^ for the three-species fit), raising the possibility that LacI molecules are present in three diffusive species, two of which are mobile (Fig. 1 *C*, *inset*, *blue* and *green dashed lines*).

To explore the influence of non-specific DNA binding and other interactions of LacI on its diffusion profile in live cells, and to explore the number of diffusive states for LacI, we examined a LacI derivative for which most of its DNA-binding domain (41 amino acids from the N-terminus) has been removed; for this mutant LacI (LacIMut), all specific, near-specific, and non-specific DNA binding modes should be non-operational. Indeed, the removal of the DNA-binding domain led to a striking change in the *D*^∗^ distribution in the absence of IPTG (Fig. 1 *D*), with LacI mobility increasing substantially. By using initially a two-species fit, we observed a large increase in the mean of the mobile fraction, with the *D*^∗^ value reaching 1.1 *μ*m^2^/s (Fig. 1 *D*, *inset*, *blue dashed line*; compare with the value of *D*^∗^ ∼ 0.4 *μ*m^2^/s observed for the full-length LacI). This increase exceeds by far the 2–3% increase in the *D*^∗^ value expected solely from the decrease in the protein molecular size due to the truncation (wild-type LacI-PAmCherry dimer, 131 kDa; LacIMut-PAmCherry dimer, 122 kDa).

Interestingly, although the LacIMut should be entirely mobile, ∼8% of tracks appear bound. This large increase in *D*^∗^ value for the LacIMut versus the mobile wild-type LacI species clearly indicates that LacI is not diffusing freely and spends considerable time bound to DNA, as suggested before (7, 12).

We also observed that the *D*^∗^ distribution is not fit as well using a two-species fit; indeed, a three-species fit (Fig. 1 *D*) shows ∼4-fold lower and more evenly distributed residuals (Fig. S2, *A* and *B*). These results argue that the single mobile species in the two-species fit actually comprises two mobile LacIMut species. The main mobile species (∼75% of all mobile molecules) has a *D*^∗^ value of ∼1.5 *μ*m^2^/s, whereas the second mobile species diffuses more slowly (*D*^∗^ ∼ 0.6 *μ*m^2^/s). The origin of the two mobile species for LacIMut is unclear, but it may reflect an equilibrium between LacI dimers and tetramers, since it is likely that a significant fraction of tetramers forms at the high LacI concentrations reached during LacI overexpression (see Discussion). We note that the fraction of immobile species is similar for the two fits (∼8% for the two-species fit and ∼6% for the three-species fit).

To study LacI mobility in the presence of its inducer (IPTG), which is expected to abolish its specific and near-specific DNA binding modes, we examined cells grown and imaged in 1 mM IPTG (Fig. 1 *E*). Once more, a three-species fit describes the data very well (Fig. S1 *H* for the residuals). As expected, the bound population decreases substantially (from ∼20% to ∼4%), and most LacI molecules become mobile, distributing between two mobile species; most molecules (∼60%) belong to the fast-diffusing population (∼0.75 *μ*m^2^/s).

Since the DNA-binding domain has been removed, the mobility of LacIMut upon addition of IPTG should remain unchanged, even though IPTG can still bind LacIMut. Consistent with expectation, there is little change in either the diffusion profile or the distribution between bound and mobile LacIMut populations after IPTG addition (Figs. 1 *F* and S2, *C* and *D*).

### The subcellular distribution of LacI in living *E. coli*

Since our single-molecule localizations provide the location of LacI molecules in the cell with 40 nm precision, we can obtain the spatial distribution of LacI from the localizations (Fig. 1 *A*). Further, to monitor the spatial distribution of the bound molecules separately from the mobile ones, we assign tracks to either bound or mobile molecules using a threshold of 0.15 *μ*m^2^ s^−1^ (22).

**Figure 2:**
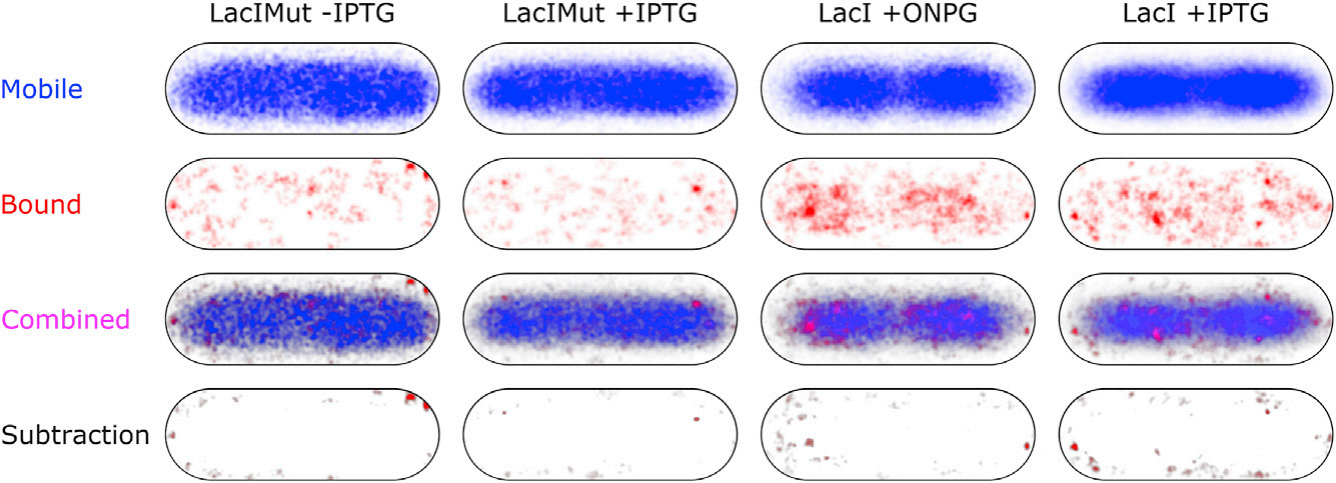
Spatial distribution of over-expressed LacI and LacIMut. Normalized histogram of LacI and LacIMut localizations with cells of length 2.5–3.5 *μ*m. Each track with five or more localizations is classified as mobile or bound. The localizations from each classified track are normalized, aggregated, and represented as a high-resolution 2D histogram. Darker regions show higher accumulation of localizations. Each cell in a row represents the type of localizations shown: only mobile, only bound, a combination of both, or a subtraction. Each column represents a different combination of strain and chemical treatment. Mobile molecules of LacIMut both with and without IPTG show bias toward the central long-axis region but don’t show a bias toward the nucleoid, in contrast to LacI with ONPG or with IPTG.

To reconstruct the probability density of LacI’s spatial distribution, we binned the classified LacI localizations into a 2D histogram (Fig. 2); we also binned and collapsed the LacI localizations along the short axis of cells after normalizing for cell width (Fig. S3; see also (25)). If a protein covers the entire cell volume uniformly, the localization probability along the short axis should resemble a “dome” (Fig. S3, *black dashed line*), since there is a higher probability that molecules of that protein will be in the mid-cell region due to the approximately cylindrical geometry of *E. coli* cells.

For LacI with all of its DNA-binding activities intact (+ONPG conditions; Fig. S3 *A*), the mobile fraction differs from the expected uniform distribution. As in the cases of RNA polymerase and nucleoid-associated protein HU (25), mobile LacI molecules (∼80% of tracks) do not distribute uniformly; instead, this effect is very likely due to constant and transient non-specific DNA binding throughout the compacted nucleoid. The bound fraction (∼20% of tracks; Fig. S3 *A*, *red line*) matches the spatial profile of the mobile population, following the distribution dictated by the nucleoid.

Under conditions where specific and near-specific DNA binding are abolished (+IPTG conditions; Fig. S3 *B*), the mobile population (∼90% of tracks) continues to follow the nucleoid; this result is consistent with the non-specific DNA-binding mode dictating the localization pattern of LacI, as it does for RNA polymerase and HU (25). However, unlike the latter two cases, the bound population (∼10% of tracks) shows a localization pattern that differs from what is expected from either nucleoid binding or a uniform distribution; instead, bound molecules appear to show bias toward the nucleoid periphery (Fig. S3 *B*, *red line*).

To dissect further the role of all DNA-binding modes in the spatial distribution of LacI, we examined the LacIMut spatial profile Although LacIMut cannot bind DNA either specifically or non-specifically, the mobile fraction under either the +IPTG or +IPTG conditions (∼90% of tracks) still showed an apparent co-localization with the nucleoid (Fig. S3, *C* and *D*, *blue curves*), mainly due to central localization bias associated with imaging fast molecules (29). This explanation is further supported by the fact that the long axis distribution of LacIMut shows no bias toward the nucleoid (Fig. 2, *Mobile*; see also Discussion). The bound fraction (which is not as well sampled as the mobile fraction) shows some exclusion from the nucleoid under +ONPG conditions, an exclusion largely abolished upon IPTG addition.

### Autofluorescent particles in *E. coli* in the absence of photoactivatable protein fusions

In control experiments with *E. coli* strains, both here and in work on electroporated molecules (30), we did observe that cells lacking genes for photoactivatable FPs or inserted labeled molecules (“autofluorescence control cells”) still carry autofluorescent particles. For all tracking-PALM and control experiments, we prepare carefully cleaned coverslips and use low-fluorescence agarose to reduce background fluorescence. In addition, we first expose cells to the excitation laser (561 nm) to bleach any background generated before we use the photoactivation laser (405 nm). Although most background fluorescence bleaches using this protocol, we have also noticed the emergence of autofluorescent particles in the sample.

**Figure 3:**
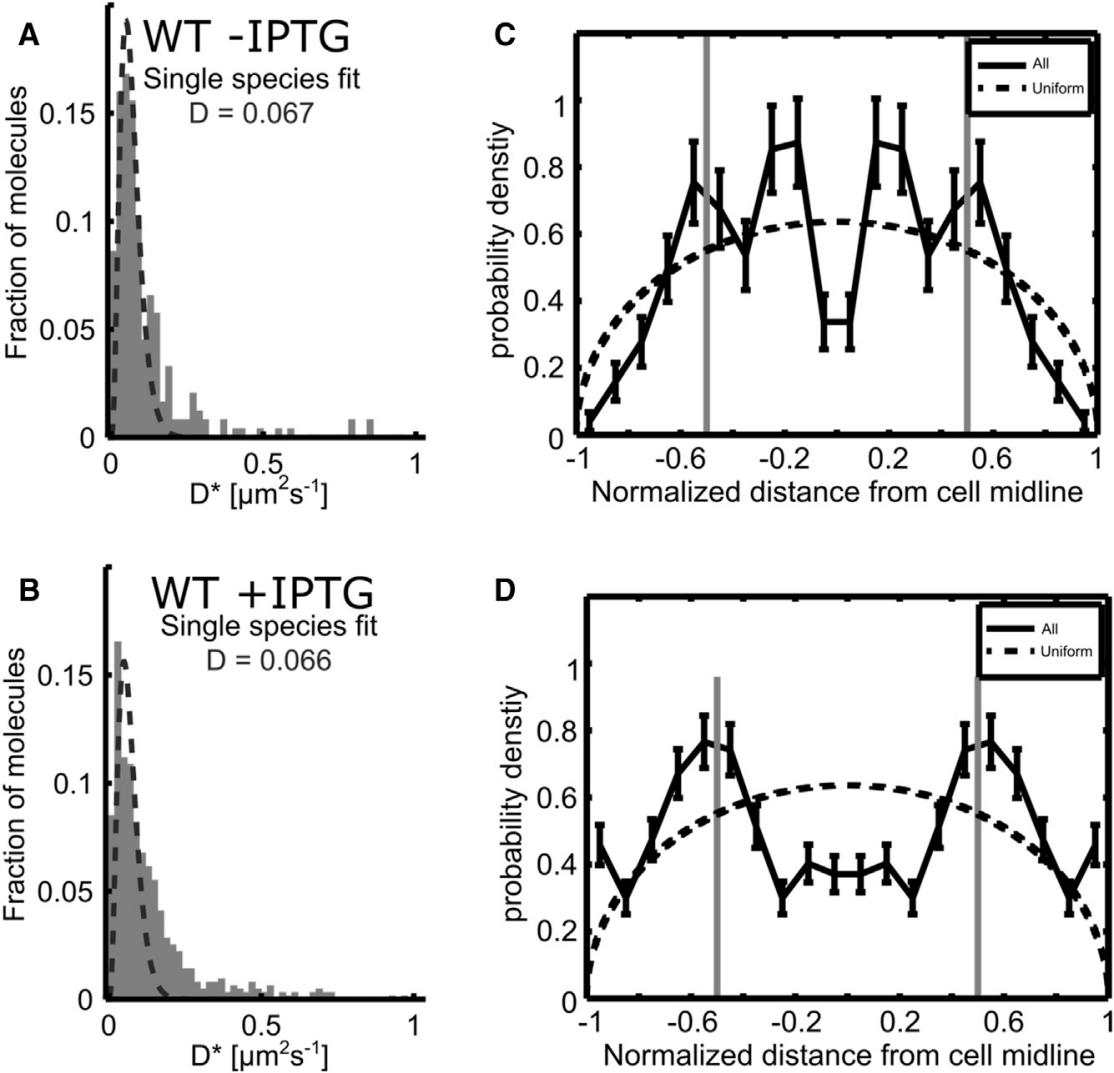
Wild-type (WT) cells with no fluorescent proteins. (*A* and *B*) Distribution of *D*^∗^ values of the background localizations found for wild-type cells without (*A*) and with (*B*) IPTG. (*C* and *D*) Spatial distribution of the localization along the short cell axis for wild-type cells without (*C*) and with (*D*) IPTG.

To account for autofluorescent particles that may distort our *D*^∗^ distribution and the spatial distribution of localizations, we tracked autofluorescent molecules (or particles) in control cells (Fig. 3, *WT cells*) that do not carry any PAmCherry fusion. Tracking-PALM studies in such cells grown in media without IPTG generated several tracks of autofluorescent molecules that appear immobile; these tracks corresponded to a narrow *D*^∗^ distribution (Fig. 3 *A*), described by a single-species distribution with a mean *D*^∗^ value of 0.066 *μ*m^2^ s^−1^. This value is lower than the 0.11 *μ*m^2^ s^−1^ obtained for immobile DNA-binding proteins (25), and not due to differences in brightness of the autofluorescent localizations (Fig. S4). In cells grown in minimal media, we find an average of ∼2 particles/cell (Fig. S5 *B*); this number could account for anywhere from 1% to 10% of tracks (considering 200 to 20 monomers per cell, respectively), although studies of proteins with large copy numbers (1000 monomers) will be unaffected. We also examined the spatial distribution of the bound tracks, which appear to be distributing rather uniformly, and show high cell-to-cell variability (Fig. 3 *A*).

To examine whether IPTG addition affects the population of autofluorescent particles, we studied autofluorescence control cells in the presence of IPTG (Fig. 3 *B*). Addition of IPTG increased the number of autofluorescent particles (Fig. S5 *B*), which reached values (∼6 molecules/cell) that need to be considered carefully for studies of low-copy-number proteins such as LacI (with ∼40 monomers/cell in unmodified *E. coli* cells). Regarding the mobility of tracks, we observed a profile similar to that without IPTG, albeit the *D*^∗^ distribution was slightly wider and the immobile particles appeared to show a more peripheral localization (Fig. 3 *B*).

To estimate the contribution of background autofluorescence to the bound fraction of LacI, we used the NoLacO + IPTG condition, where we expect to see little LacI binding; we observe ∼40 molecules for 2.5- to 3.5-*μ*m cells (Fig. S5 *C*), of which ∼30% (∼12 molecules) are immobile. Under the same conditions (i.e., +IPTG), we find ∼7 autofluorescent molecules in wild-type cells, which are almost exclusively immobile. This means that more than half (∼18% out of the total ∼30%) of the bound fraction for NoLacO + IPTG can be accounted for by autofluorescent particles.

### The in vivo diffusion profile of LacI expressed at native copy numbers

We then measured the LacI mobility and spatial distribution in two *E. coli* strains carrying wild-type copy numbers for LacI. Both strains had a chromosomal copy of full-length LacI fused to PAmCherry and carried no plasmid for LacI overexpression. In both strains, the wild-type operators (lacO1, lacO2, and lacO3) have been deleted, resulting in a fully induced expression of LacI with or without IPTG. The first strain (named “6xlacO”) has six proximal consensus *lac* operators (a 21-bp DNA sequence) within a 194-bp DNA segment (Fig. S6); the second strain (named “NolacO”) contains no *lac* operators. The strains allow us to observe a slightly higher amount of LacI binding to operators (up to 12 monomers, all of high affinity) compared to the wild-type (six monomers, with differing affinities).

**Figure 4:**
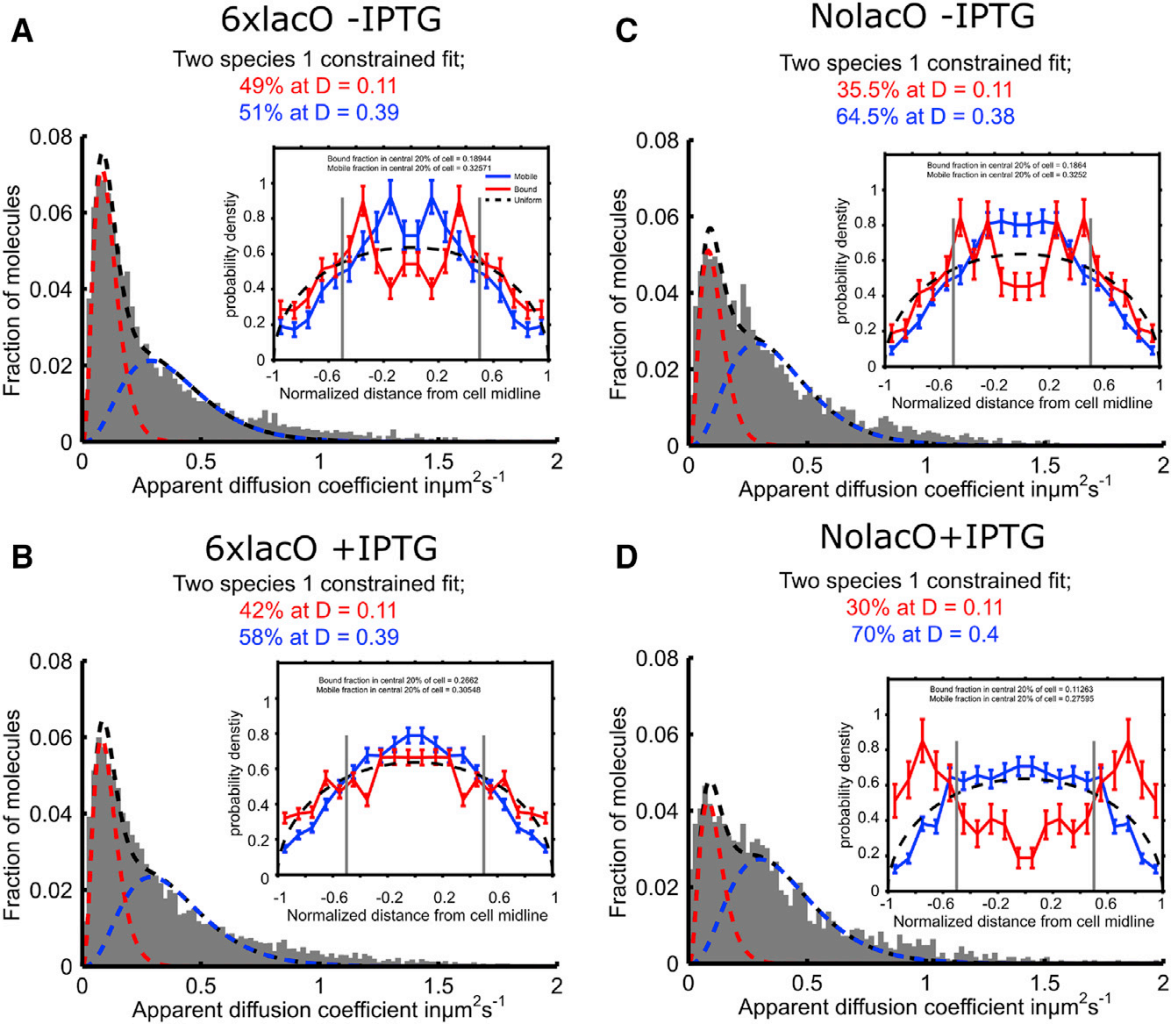
Diffusion profile and spatial distribution of Lacl in strains with native copy numbers. We tracked LacI-PAmCherry with and without a 1 mM IPTG treatment using the 6xlacO and NolacO strains in live cells. (*A*–*D*) Diffusive behavior from the calculated apparent diffusion of each track, displayed as the outline of the histogram of all the tracks. The insets show the spatial distribution for each data set.

We first tracked LacI in the 6xlacO strain in the absence of IPTG; in this case, all DNA binding modes are operational, which should reduce mobility for most LacI molecules. Consistent with this, we observed that ∼50% of the tracks were bound (Fig. 4 *A*); further, the mobile fraction (as reflected in its *D*^∗^ distribution) was very similar to that from the overexpressed sample (0.39 vs. 0.41 *μ*m^2^/s, respectively). We also examined the LacI spatial distribution; due to the low copy number of LacI in these cells (Fig. S5 *C*), the distribution was noisier than for over-expressed LacI; we thus analyzed our distributions to characterize the bound fraction and a single mobile species. The mobile distribution (Fig. 4 *A*, *inset*) resembles that of over-expressed LacI, i.e., preferential localization within the bacterial nucleoid. By contrast, the bound localizations appear to show a preference for the nucleoid periphery and are less abundant in the central region, although this may be biased due to the distribution of immobile autofluorescent particles (see Discussion).

We also examined the effect of IPTG addition on the 6xlacO strain, where the LacI molecules should dissociate from the six tandem operators, decreasing overall LacI binding. Consistent with expectations, the bound fraction decreased to ∼40% (Fig. 4 *B*); the increased mobile fraction still showed mobility that matched that of the mobile fraction in 6xlacO without IPTG (with a mean *D*^∗^ value of 0.39), as in the case of LacI over-expressed from a plasmid in the presence of ONPG. The mobile fraction once again matched the nucleoid spatial distribution, whereas the bound fraction appeared uniformly distributed (Fig. 4 *B*, *inset*).

To further study the modes of LacI binding in vivo, we examined the NolacO strain, which contains no natural operators. When LacI has no operators and IPTG is absent, LacI can still bind non-operator DNA sites, such as near-specific sequences (which may exist at various chromosomal loci) and non-specific DNA; however, specific binding (such as that for the 6xlacO strain) will be absent. Consistent with this, there is a decrease in the bound fraction (from ∼50% for the 6xlacO strain without IPTG, to ∼35% for the NolacO strain without IPTG), albeit with little change in spatial localization; further, the mobile fraction is similar in mobility and spatial localization to that seen for the 6xlacO strain.

Finally, we examined how the LacI profile in NolacO is affected by IPTG, which is expected to remove all specific and near-specific binding and to reduce non-specific binding (31). Indeed, IPTG addition (Fig. 4 *D*) reduces the bound fraction to ∼30%, and shows localizations that imply some exclusion from the nucleoid; however, at these low copy numbers, the presence of autofluorescent background particles may skew the data. The mobile fraction shows mobility essentially identical to that for 6xlacO ±IPTG and NolacO −IPTG, reinforcing the conclusion that LacI mobility reflects a combination of 3D diffusion interrupted by non-specific binding to the bacterial nucleoid.

### Bound LacI associates with the expected chromosomal locus

To ensure that the bound molecules indeed associate with the desired chromosomal location, and to examine the presence of any LacI concentration gradient in living *E. coli*, we examined the location of the LacI molecules relative to the actual location of the six *lac* operators within the cell. To label the chromosomal position of the 6xlacO tandem operators, we constructed a strain that carries a FROS marker (2, 3) based on MalI (Fig. S7), a transcriptional repressor that regulates maltose metabolism and transport. We placed 20 MalI operator sites near the six *lac* operators (with the boundaries of the two tandem operator systems separated by 17 bp; Fig. S7) and expressed MalI-GFP from a plasmid; binding of the MalI molecules to their operators forms a single fluorescent spot per labeled genetic region, allowing us to check the number of copies and location of these genes in individual cells. If LacI molecules bind to the 6xlacO operators, the LacI localizations should cluster near the FROS marker; further, LacI clustering should disappear upon IPTG treatment.

**Figure 5:**
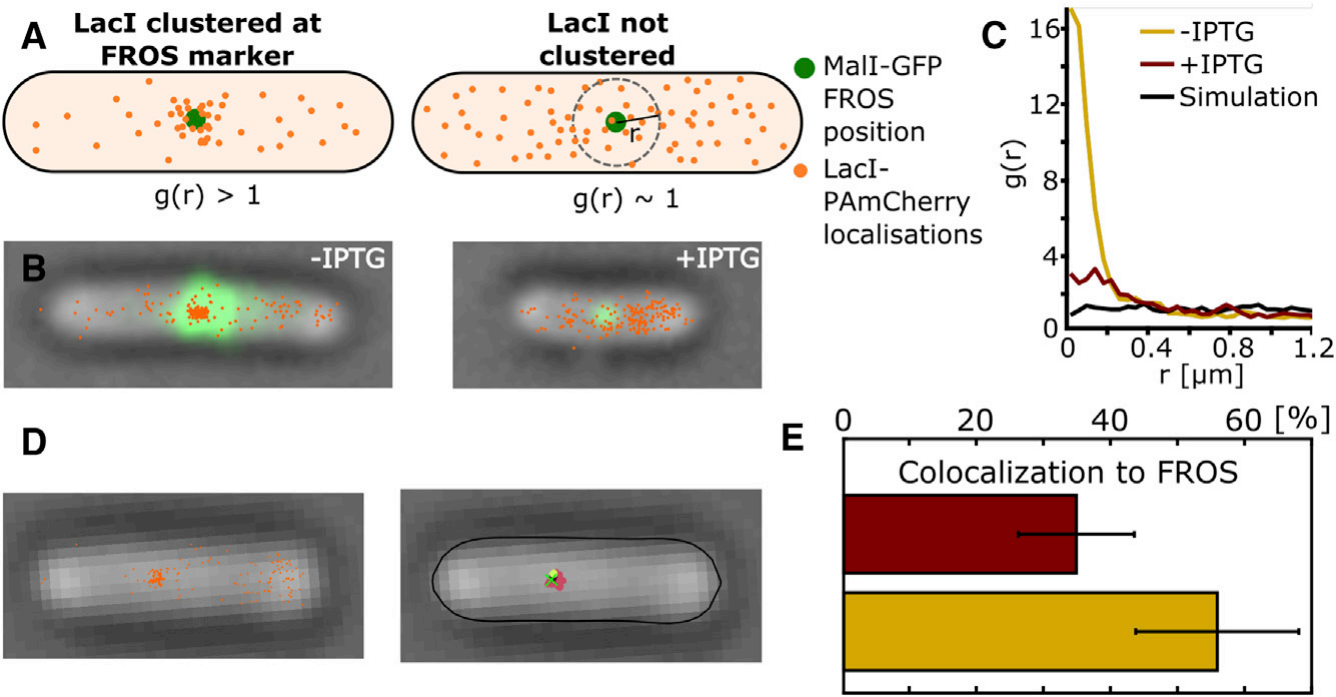
Clustering of LacI-PAmCherry near the FROS marker. We calculated the radial distribution function to determine the clustering of the LacI near its binding site by introducing an array of 20 tandem operators of MalI adjacent to the LacI operator sites. To label the MalI operators, MalI-GFP is expressed from a plasmid. (*A*) Clustered localizations will have a radial distribution, *g*(*r*), of >1; unclustered localizations randomly distributed in the volume of the cell will yield *g*(*r*) ∼ 1. (*B*) Experimental data for the FROS marker are shown along with the identified localizations of the LacI-PAmCherry. (*C*) Radial distribution functions are shown for the 6xlacO strain with and without addition of IPTG. The black line plot close to *g*(*r*) = 1 represents random simulated positions, with a number of localizations identical to real data found in the cells. (*D*) Localizations for a different cell are shown in the left image, and the results from the clustering algorithm in the right image (clusters of different colors; the green cross is the localization of the FROS marker). (*E*) Fractions of clusters successfully co-localized to a FROS localization.

After imaging the FROS foci to obtain the relative subcellular position of the 6×lacO sequence (Fig. 5 *A*), and subsequent tracking PALM on LacI-PAmCherry in the same cells, we noticed that, as expected, many LacI localizations were proximal to the FROS marker in the absence of IPTG (Fig. 5 *B*). To quantify the LacI clustering relative to its operator sites, we applied the radial distribution function analysis, *g*(*r*) (32, 33), to our PALM data. For each cell, the distance between the FROS marker and all LacI localizations is determined. To account for the geometry of *E. coli* cells, we generated simulated random localizations within the cell (using a uniform molecular density in the cell) and determined the distances of the simulated localizations to the LacO sites. The radial distribution is calculated as the ratio of experimentally acquired distances to simulated distances. Thus, randomly distributed data yield a *g*(*r*) of 1, whereas clustered data yield *g*(*r*) values >1.

By calculating the radial distribution functions for the 6xlacO strain (Fig. 5 *C*); we found that the LacI localization exhibits non-random behavior in both untreated and IPTG-treated cells. In untreated cells, the probability of LacI residing near its operators (Fig. 5 *C*, *yellow line*) is up to 16-fold higher than would be expected for a simulated random distribution (Fig. 5 *C*, *black line*), clearly indicating that LacI specifically binds to its tandem operators. In contrast, in IPTG-treated cells, this probability is only slightly higher than random (Fig. 5 *C*, *red line*). The LacIs in cells without IPTG were 11 times more likely to be found within a radius of 200 nm of the FROS position (Fig. 5 *B*, *green area*); in the treated cells, this was only 2.7 times more likely. The main reason for *g*(*r*) > 1 for treated cells is that FROS markers are located on the nucleoid and are distributed more centrally (Fig. S9); as such, we also expect a higher-than-random probability of finding mobile LacI molecules near the FROS marker (even when IPTG is added).

The characteristic length dependence of *g*(*r*) in our experiments with untreated cells containing LacI is attributed to several factors: the localization precision for the FROS marker and for the PALM localizations; the LacI confinement to the nucleoid (due to non-specific binding); the higher probability of finding a molecule closer to the cell center (due to time averaging within a frame, i.e., “localization centralization”); and the 2D projection of the 3D separation between FROS markers and lacO sites (due to their separation by ∼20–800 bp within the chromosome). Indeed, the *g*(*r*) decay shows an increased probability in the 0–200 nm region, reflecting mainly our localization precision of ∼40 nm plus the distance from the FROS marker sites to the lacO sites.

Using the co-localization from the radial distribution function (200 nm), we counted the number of molecules within a certain distance from the FROS marker (Fig. S8); we calculated ∼5.8 monomers of LacI co-localized to the FROS marker in the absence of IPTG (Fig. S8, *blue line* at 200 nm), whereas, in the presence of IPTG this number decreased to ∼3 (Fig. S8, *red line* at 200 nm).

We also tested our ability to identify native operator regions using native copy numbers. In wild-type cells, operators may appear in close proximity, such as for the three native *lac* operator regions linked to the *lac* operon. Finding regions of clustering may help us deduce regions of activity. In essence, this parallels a typical FROS experiment, where a cluster of bound proteins can be distinguished from the free diffusing proteins, which appear as diffuse background. Unlike native operators, FROS markers can be tailored to increase their detection efficiency by adding any number of operators necessary.

To test our ability to identify tandem operators using tracking PALM, we clustered the LacI localizations (Fig. 5 *D*) and checked whether the cluster is real by examining the co-localization of LacI clusters with the MalI FROS markers. We determined the position of the cluster relative to the nearest FROS marker (using the 200 nm threshold) and estimated the fraction of clusters that co-localize with a FROS with and without IPTG (Fig. 5 *E*). We find that without IPTG, ∼60% of the clusters co-localize to a FROS position, whereas with addition of IPTG, ∼30% of clusters co-localize to a FROS (see also Fig. S9 for the overall subcellular distribution of our FROS markers). These results showed that we can detect a locus-specific signal even for TFs with very few copies binding to DNA, and they led to recommendations on how to improve the locus detection efficiency (see Discussion).

## Discussion

Around 60% of TFs in *E. coli* exist in the cell in low- to medium-copy numbers (1–100 monomers (34)). Due to its sensitivity, quantitative nature, and ability to evaluate intracellular mobility of single molecules, PALM is one of the best methods to study protein function and spatial distribution in vivo. Here, we used tracking PALM to study the mobility and DNA interactions of the *lac* repressor; these studies provide guidelines on using tracking PALM to study low-copy number DNA-binding proteins in vivo.

LacI and other TFs can bind DNA in three main modes of interaction: a specific, a near-specific, and a non-specific mode. In wild-type *E. coli*, and in the absence of lactose, LacI can bind specifically to three operator sites (lacO1, lacO2, and lacO3), as well as to multiple sites for which it has near-specific binding. In the presence of lactose, LacI cannot bind specifically or near specifically. In our work, we set out strategies to dissect these different modes of interaction with DNA by performing tracking PALM in cells that over-express LacI, and in cells containing LacI in its native copy numbers.

### Over-expressing LacI provides a clear view of non-specific LacI-DNA interactions

LacI over-expression (>200 monomers/cell) addresses the main problems associated with native numbers, since it provides high statistics per cell and minimizes the effect of background; further, over-expression allows us to focus more on the non-specific mode of the LacI-DNA interactions, since all native *lac* operators and any near-specific sites are fully occupied by a small fraction of over-expressed LacI, whereas the majority of LacI molecules search for sites via a combination of 1D sliding and 3D diffusion. This was the case for LacI over-expressed in ONPG: ∼25% of LacI was bound (reflecting specific and near-specific DNA interactions), whereas the remaining ∼75% was mobile, with a mean *D*^∗^ value of 0.40 *μ*m^2^/s.

As with many other DNA-binding proteins, LacI searches for sites of function by combining DNA binding with 3D diffusion, with the LacI diffusion profiles yielding the fraction of time spent diffusing versus being bound to DNA. To achieve this, one needs to track LacI diffusion devoid of any interactions with DNA, either by imaging DNA-free regions from filamentous cells (25) or by removing the DNA-binding domain of the protein (7). Using the second approach, we studied a LacI derivative lacking the DNA-binding domain; this DNA-binding mutant diffused much faster than LacI, and was unaffected by the presence of IPTG.

Our results also suggested the presence of two mobile species for the over-expressed LacIMut. What gives rise to such species for both wild-type and mutant lacI? Since LacIMut lacks DNA binding, it is unlikely that the mobility difference is due to different LacImut-DNA interactions. Instead, we favor the possibility of in vivo LacI tetramerization despite its C-terminal labeling with PAmCherry. Published work using LacI-Venus C-terminal fusions (7) had suggested that such fusions bind as a dimer when expressed at low concentration in *E. coli*; however, the same report indicated (see Fig. S1 in (7)) that even at low concentrations (∼7 LacI monomers per *lacI* gene), there is a significant amount of tetramer present. In our LacI-over-expessing strains, the LacI concentration is much higher (>500 LacI monomer molecules/cell), so LacI tetramerization will be promoted even further. We also expect significant tetramerization at the native copy numbers (∼40 LacI monomers).

In the presence of IPTG, LacI no longer binds specifically or near-specifically; this condition allowed us to focus on the diffusion and DNA interactions of the LacI-IPTG complex. Indeed, >90% of the LacI molecules were mobile. Our results also establish that little FP-driven aggregation is present under our over-expression conditions, since the fraction of immobile (or very slowly diffusing) molecules is <10%.

Intriguingly, the LacI-IPTG complex interacted with the DNA for a shorter fraction of time, as evident from its faster diffusion (*D*^∗^ = 0.53 *μ*m^2^/s) relative to LacI in the absence of IPTG. The decreased affinity of IPTG-bound LacI toward non-specific DNA has been shown before (31), and it suggests that un-complexed LacI spends, on average, 5% more time on DNA than the LacI-IPTG complex.

### Diffusion analysis of LacI in native copy numbers

To study the different modes of LacI-DNA interaction in the context of the native LacI copy numbers, we studied the diffusion profile of LacI in strain 6xlacO, where specific and near-specific DNA binding should dominate LacI-DNA interactions in the absence of IPTG. Indeed, we observed that ∼50% of the tracked molecules were bound in the absence of IPTG, with the addition of IPTG reducing this amount to ∼40%. We attribute the difference of 10% to the expected loss of *specific* binding, whereas the remaining binding reflects mainly molecules mis-classified as bound (due to the short duration of the trajectories and their Brownian nature and to the fitting of closely spaced distributions), background localizations.

We also examined a strain without *lac* operators (NolacO), which is key in studying near-specific interactions. The decrease in the bound fraction (35–30%) upon IPTG addition provides direct evidence for the presence of near-specific LacI binding sites other than the three well-known *lac* operators. Further, the comparison with the 6xlacO strain shows that ∼40% of the bound fraction is due to specific and near-specific binding, with the rest accounted for by background localizations and mis-classified Brownian trajectories (the latter accounting for ∼1.3% of all tracks, based on diffusion simulations; see Fig. S10). As a result, background localizations set a limit on our ability to apply tracking PALM to studies of low-copy-number proteins.

Finally, the number of molecules detected in the two strains with native copy numbers (∼50 monomers) is within ∼30% of the literature value, showing that any factors that complicate accurate copy-number quantification (blinking; the presence of mis-folded, non-matured, or pre-bleached fluorescent fusions; or lost localization due to noise) do not lead to large deviations in our study.

### The spatial profile of LacI shows that mobile LacI co-localizes with the nucleoid

In addition to the diffusion work, we observe the spatial distribution of LacI. In both over-expressed and native copy numbers, mobile LacI shows a bias toward the nucleoid. This provides further confirmation that LacI interacts extensively with the DNA while searching for its targets (7).

The spatial profile of LacIMut across the cell short axis also seemed to follow the nucleoid; however, close examination of the long-axis distribution showed that LacIMut does not follow the double-nucleoid shape as LacI does (Fig. 2). We attribute this spatial profile to our exposure time relative to the fast diffusion of LacIMut (note: *D*^∗^_LacIMut_ > *D*^∗^_LacI_); at this timescale, LacIMut diffuses a large distance, biasing the center of the image of tracked molecules toward the center of the cell (with the cell wall acting as a border). The spatial profile for LacI and LacIMut was further complicated by background fluorescence particles, which appeared to be excluded from the nucleoid (see next section).

### Challenges with low to moderate copy numbers using PALM

While working with low (1–5 monomers) to moderate (5–100 monomers) copy numbers, any background particles will have a larger effect on the data, and the number of classifiable tracks per cell will be small. We found that most autofluorescent background particles are immobile, thus increasing the bound fraction of tracks when the copy numbers of the protein of interest are low. Since it is the bound fraction that is usually linked with function (e.g., repression in the case of LacI, DNA synthesis for DNA polymerase I, or transcription for RNA polymerase), it is important to assess the effect of background tracks. Background particles will affect the spatial distribution of the classified tracks, since they seem excluded from the nucleoid volume, affecting the distribution of the bound tracks. In addition, aggregation phenomena have been reported for PAmCherry fusions, which may also contribute to the observed background (35). This calls for caution in interpreting the results and for careful control experiments with wild-type cells.

### Identifying small clusters of DNA-binding sites using tracking PALM

TFs frequently bind multiple specific sites in proximity to one other, as in the case of the *lac* operon for LacI and the L-arabinose operon for the AraC regulator. To probe our ability to identify such multi-site regions purely from tracking-PALM data, we examined the co-localization of LacI clusters with a marked chromosomal locus (Fig. 5). We found that we could identify a region with six operators using tracking PALM, but with many false positives and false negatives: ∼60% of the clusters co-localized with the tagged chromosomal locus. This estimate gives us an approximation dependent on localizing both the FROS marker and the cluster. False positives include background particles yielding immobile localization that may co-localize with our chromosomal marker, as well as blinking single particles. False negatives may be caused by inability to collect enough localizations from the multi-operator region to form a cluster; this may be due to having too few bound photoactivatable proteins (e.g., we are unsure whether the 6xlacO region is fully occupied), not detecting or not photo-activating all the PAmCherry-labeled LacI molecules, or not detecting all the MalI FROS foci.

The issues addressed in our work are common in tracking low- to moderate-copy-number proteins. Our approach and insights will help in the study of TFs or other low-copy-number proteins in bacteria, as well as in developing experimental and computational approaches that improve our ability to work with this important class of DNA-binding proteins.

## Author Contributions

A.N.K., S.J.W.B., and F.G.d.L. designed research; F.G.d.L. and L.S. performed research; M.S. contributed analytic tools; F.G.d.L. and M.S. analyzed data; and A.N.K. and F.G.d.L. wrote the article.

## Supporting Material

Document S1. Supporting Materials and Methods and Figs. S1–S10

Document S2. Article plus Supporting Material
