# Supplementary material for: Tracking Low-Copy Transcription Factors in Living Bacteria: The Case of the lac Repressor
Source: Biophys J. 2017 Apr 11;112(7):1316–27. doi: 10.1016/j.bpj.2017.02.028 (PMC5390046; doi:10.1016/j.bpj.2017.02.028)
Supplement: Document S1. Supporting Materials and Methods and Figs. S1–S10 [file mmc1.pdf]

Biophysical Journal, Volume *112*

Supplemental Information

Tracking Low-Copy Transcription Factors in Living Bacteria: The Case of the *lac* Repressor

Federico Garza de Leon, Laura Sellars, Mathew Stracy, Stephen J.W. Busby, and Achillefs N. Kapanidis

METHODS

Strain Construction. Strains were constructed as published in (Lee et al., 2009). 6 LacI operator DNA sites (5'_-AATTGTGAGCGGATAACAATT-3') were inserted adjacent to the araBAD promoter in a strain that also had a chromosomal LacI::GFP fusion, LR06.

6x lac operator sequence (lacI Binding sites are in bold typeface):
GAATTCGGATCCCTCGAGCCAGCACGTAGCTAGCAGAGAGTAAGGAATTGTGAGCGGA
TAACAATTAAAAGTCAGAAATTGTGAGCGGATAACAATTAAAGACGGAGAATTGTGAGC
GGATAACAATTAATAAAGCAGCTCTAGCATCAACGCGCAAATTGTGAGCGGATAACAAT
TAGCAAGACACAATTGTGAGCGGATAACAATTCTGGATAGGCAATTGTGAGCGGATAAC
AATTCACAGCGCCCCTCTAGAGTCGACAGATCTAAGCTT

6x lac operator sequence + 20x MalI operator sequence (lacI Binding sites are in bold typeface; MalI sites are italicized; restriction sites are underlined):

XhoI EcoRI SacI KpnI XmaI BamHI XbaI

CTCGAGGAATTCGAGCTCGGTA**CCGGGG**ATCCTCTAGAGTCT*GATAAACGTTTATC*AC

GTGTCGAGCATG*GATAAACGTTTATC*GCTAGAGTCT*GATAAAACGTTTTATC*ACGTGTC

GAGCATG*GATAAAACGTTTATC*GCTAGAGTCT*GATAAAACGTTTTATC*ACGTGTCGAGC

ATG*GATAAAACGTTTTATC*GCTAGAGTCCT*GATAAAACGTTTTATC*ACGTGTCGAGCATG

*GATAAAACGTTTTATC*GCTAGAGTCCT*GATAAAACGTTTTATC*ACGTGTCGAGCATG*GAT*

*AAAACGTTTTATC*GCTAGAGTCCT*GATAAAACGTTTTATC*ACGTGTCGAGCATG*GATAAA*

*ACGTTTTATC*GCTAGAGTCCTGATAAAACGTTTTATCACGTGTCGAGCATG*GATAAAAC*

*GTTTTATC*GCTAGAGTCCT*GATAAAACGTTTTATC*ACGTGTCGAGCATG*GATAAAACGTT*

*TTATC*GCTAGAGTCCT*GATAAAACGTTTTATC*ACGTGTCGAGCATG*GATAAAACGTTTTA*

NheI

*TC*GCTAGAGTCCT*GATAAAACGTTTTATC*ACGTGTCGAGCATG*GATAAAACGTTTTATC*G

CTAGAGGGGCGCTGTG**AATTGTTATCCGCTCACAATT**GCCTATCCAG**AATTGTTATCC**

GCTCACAATTGTGTCTTGCT**AATTGTTATCCGCTCACAATT**TGCGCGTTGATGCTAGAG

CTGCTTTATT**AATTGTTATCCGCTCACAATT**CTCCGTCTTT**AATTGTTATCCGCTCACAA**

TTTCTGACTTTT**AATTGTTATCCGCTCACAATT**CCTTACTCTCTGCTAGC

SUPPLEMENTAL FIGURES

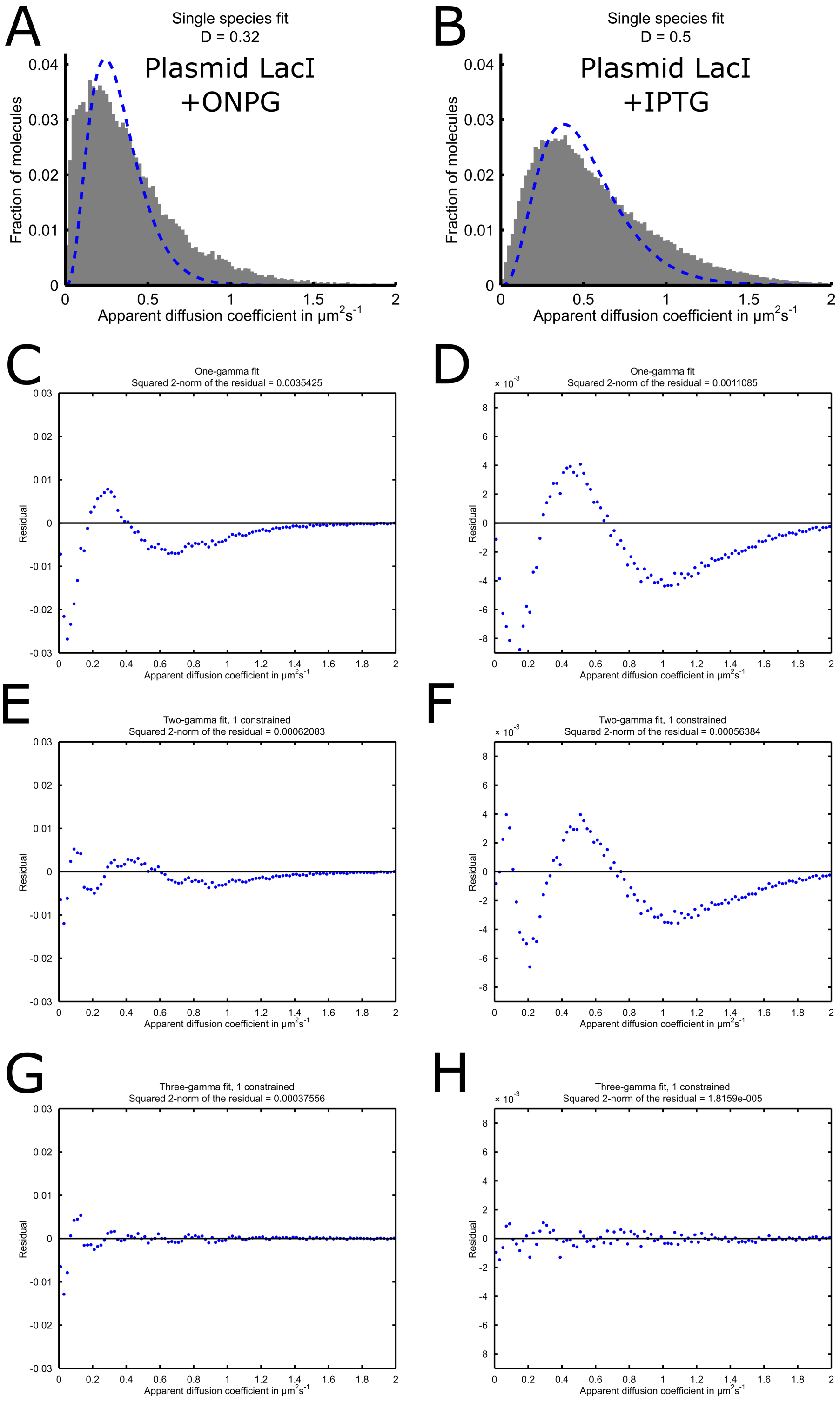

Figure S1. Single-species fits for plasmid-encoded LacI. Using the histograms from Fig. 1 for the LacI expressed from a plasmid in two cases: in the presence of ONPG (A, residuals of left column) and in the presence of IPTG (B, residuals of right column). The significant deviations from of the fits from the D* distributions show that a single-fit cannot describe the D* distribution well. The residuals for the one-gamma fit are shown in C-D. The progressive improvement with increasing use of gamma functions to fit the data is shown in E-F for a two-gamma fit and G-H for a three-gamma fit.

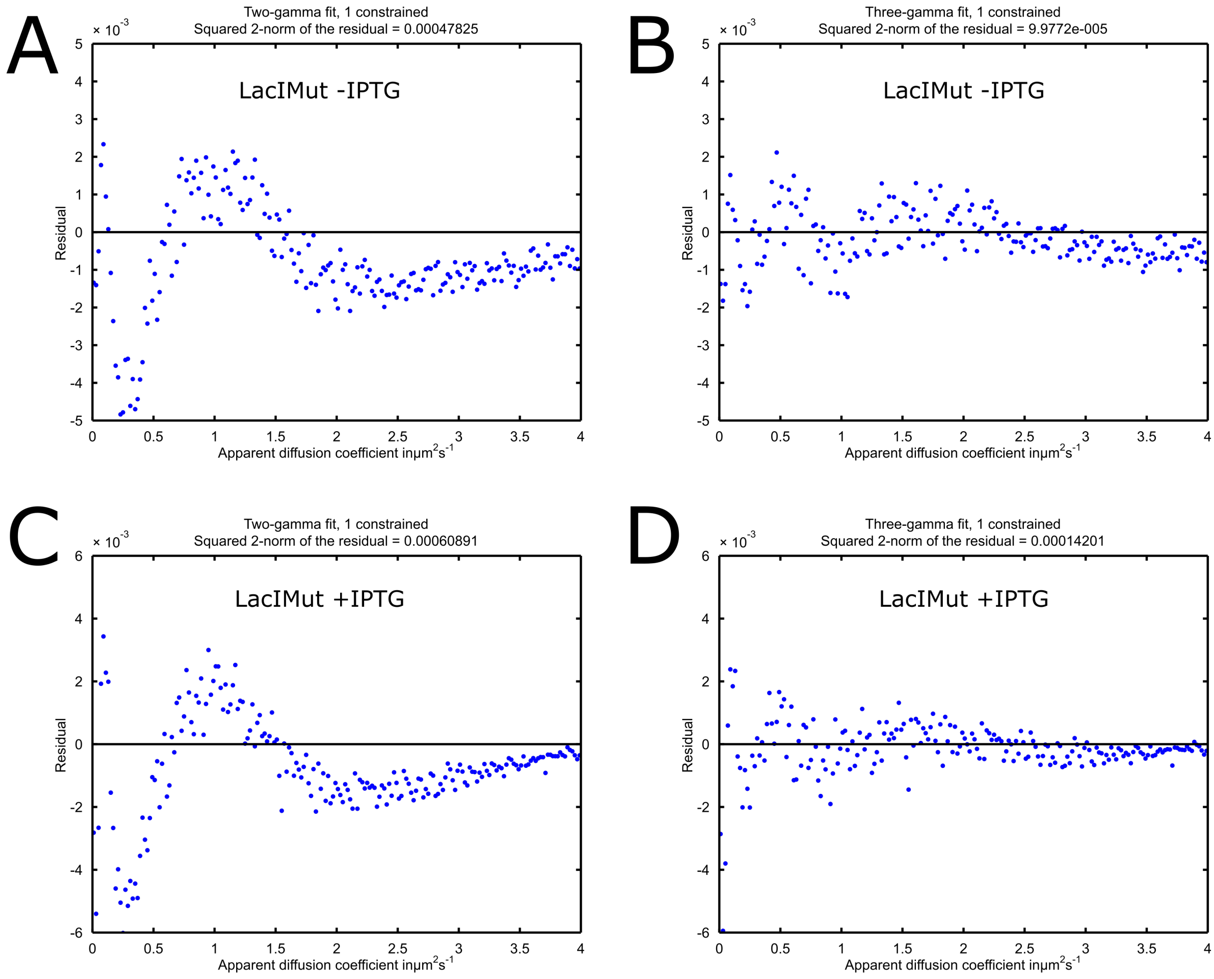

Figure S2. Residuals for the two-gamma and three-gamma fits of LacIMut. The residuals for the fits from Fig. 1 are shown. The fit of LacIMut in absence of IPTG improves from the two-gamma function (A) to the three-gamma function (B), as shown by the reduced squared residual and the lower bias in the residual. A similar case is observed in the presence of IPTG, where we see a marked improvement when going from the two-gamma function (C) to the three-gamma function (D).

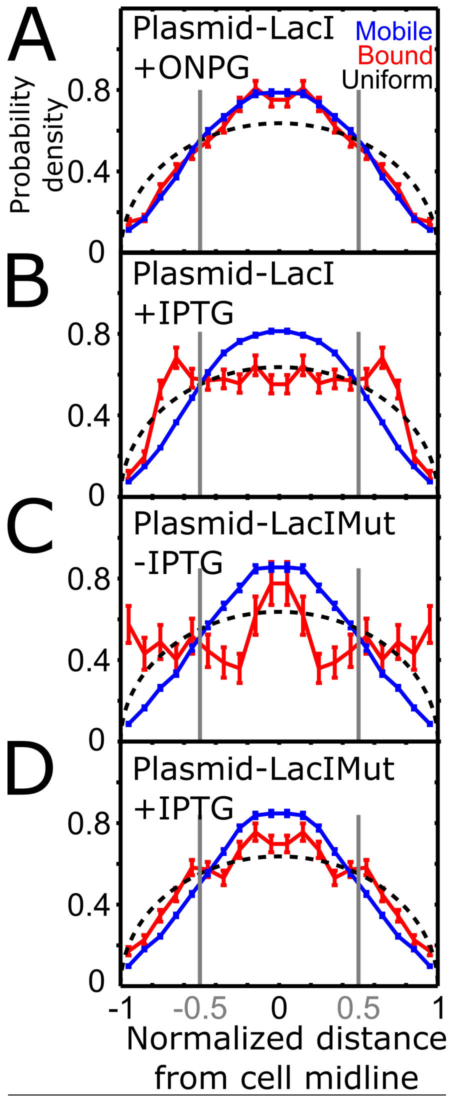

Figure S3. Spatial distribution of the localization along the short cell axis. The short axis distribution of the molecules is measured with −1 and 1 representing the cell membrane in the normalized distance. Because of their single nucleoid, between 1.5 μm and 2.5 μm long cells were used to generate the plots. Error bars represent the error of a Poisson distribution (+/- normalized $\sqrt{N}$, N being the bin count number). (A-B) represent the spatial distributions of the samples analyzed in Fig. 1C-F.

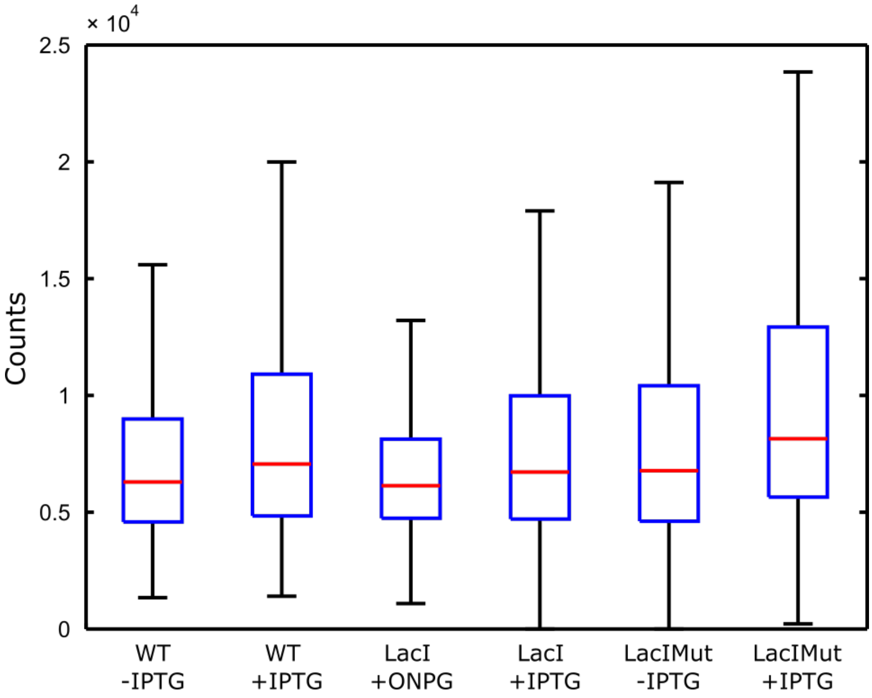

Figure S4. Fluorescence intensity corresponding to the localizations for each experiment. A 7 by 7 pixel window is used to fit a 2D Gaussian and the total number of counts under the area of the Gaussian calculated. The red line represents the median of the data, whilst the blue box encloses 50% of the data points. The whiskers represent either the minimum or maximum values.

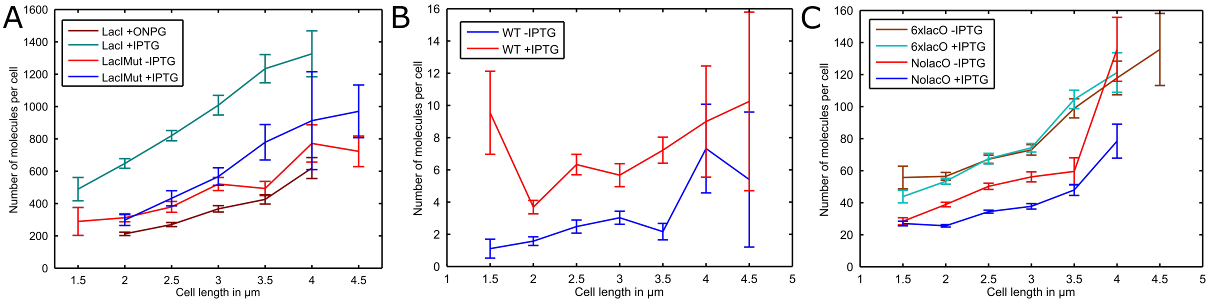

Figure S5. Copy numbers per cell length. A) Copy numbers of plasmid-expressed LacI expressed (+ONPG and +IPTG) and for LacIMut (±IPTG). B) Copy numbers for the wild-type cells shown with and without IPTG. C) Copy numbers for the chromosomally modified strains 6xlacO and NolacO (±IPTG). Error bars: SEM.

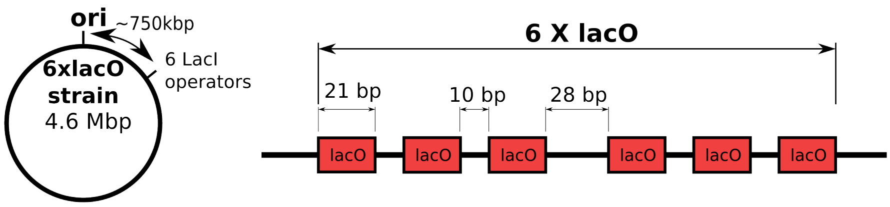

Figure S6. Description of the 6xlacO and NolacO strains. In 6xlacO, 6 tandem lac operators are inserted into the genome and the native operators removed. In NolacO, there are no operators in the genome. For the sequence, see supplement (*Strain Construction)*.

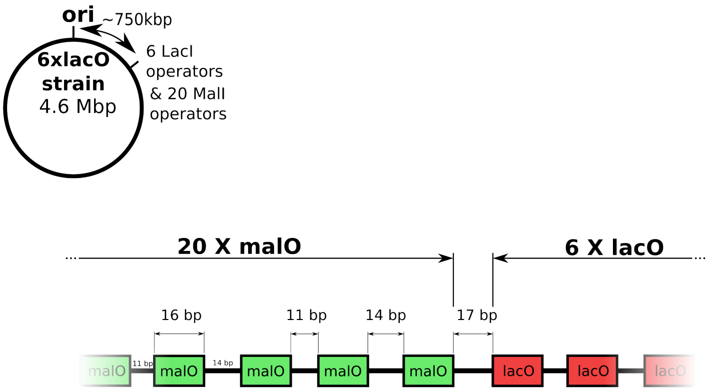

Figure S7. Description of 6xlacO strain with 20 Mal operators. To generate a MalI based FROS marker, we added 20-MalI operators next to the 6 *lac* operators from the 6xlacO. For the sequence, see supplement (*Strain Construction)*.

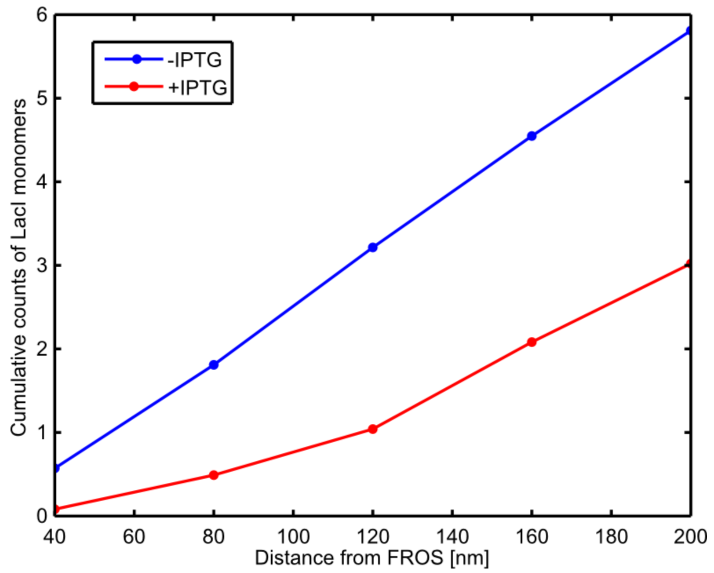

Figure S8. Cumulative counts of the of the LacI monomer from different distances to the FROS marker. Each point represents the number of counts for the LacI monomers found within a distance from the FROS marker. In the case for –IPTG, 5.8 monomers where found to be within 200nm of the FROS foci on average and for +IPTG 3.0 monomers.

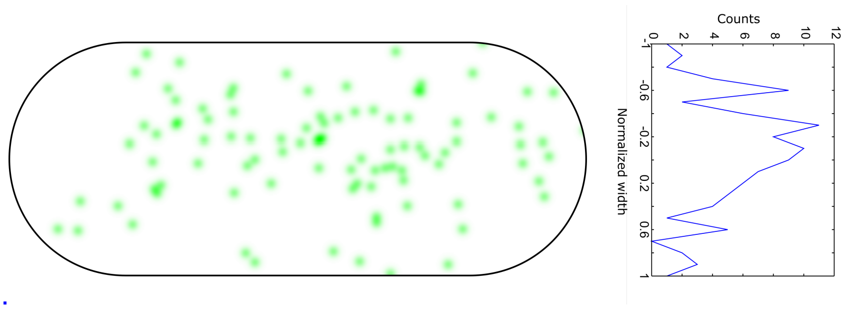

Figure S9. Spatial distribution of the FROS marker. One localization is identified per FROS marker and normalized. The DNA FROS loci appear to localize closer to the long axis of the cell, and do not avoid the cell center.

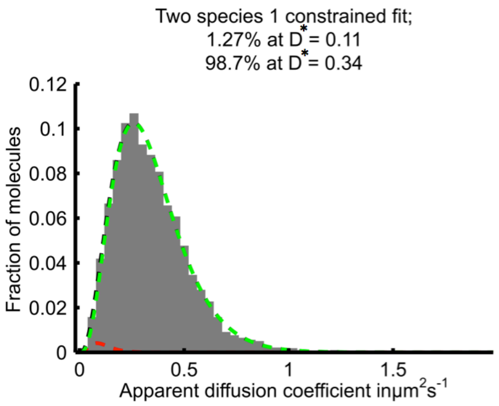

Figure S10. Simulated tracks and classification through fitting. We simulated molecules diffusing at D=0.4 μm^2/s in 500 cells with 40 molecules each. Each cell has 2.9 μm length and 0.9 μm width, and localization error is simulated to 44 nm. Using 2-species fitting, the fraction of misclassified is ~1.3%, which accounts for a very small fraction of apparently immobile molecules in the NoLacO background.
